# Gut microbiota as a biomarker and modulator of anti-tumor immunotherapy outcomes

**DOI:** 10.3389/fimmu.2024.1471273

**Published:** 2024-11-28

**Authors:** Jiexi Yan, Lu Yang, Qingmiao Ren, Chan Zhu, Haiyun Du, Zhouyu Wang, Yaya Qi, Xiaohong Xian, Dongsheng Chen

**Affiliations:** ^1^ The Precision Medicine Laboratory, The First Hospital of Lanzhou University, Lanzhou, Gansu, China; ^2^ The State Key Laboratory of Neurology and Oncology Drug Development, Jiangsu Simcere Diagnostics Co., Ltd., Nanjing Simcere Medical Laboratory Science Co., Ltd., Nanjing, Jiangsu, China

**Keywords:** immune-checkpoint inhibitors, gut microbiota, therapeutic efficacy, microbiota-based interventions, immunotherapy

## Abstract

Although immune-checkpoint inhibitors (ICIs) have significantly improved cancer treatment, their effectiveness is limited by primary or acquired resistance in many patients. The gut microbiota, through its production of metabolites and regulation of immune cell functions, plays a vital role in maintaining immune balance and influencing the response to cancer immunotherapies. This review highlights evidence linking specific gut microbial characteristics to increased therapeutic efficacy in a variety of cancers, such as gastrointestinal cancers, melanoma, lung cancer, urinary system cancers, and reproductive system cancers, suggesting the gut microbiota’s potential as a predictive biomarker for ICI responsiveness. It also explores the possibility of enhancing ICI effectiveness through fecal microbiota transplantation, probiotics, prebiotics, synbiotics, postbiotics, and dietary modifications. Moreover, the review underscores the need for extensive randomized controlled trials to confirm the gut microbiota’s predictive value and to establish guidelines for microbiota-targeted interventions in immunotherapy. In summary, the article suggests that a balanced gut microbiota is key to maximizing immunotherapy benefits and calls for further research to optimize microbiota modulation strategies for cancer treatment. It advocates for a deeper comprehension of the complex interactions between gut microbiota, host immunity, and cancer therapy, aiming for more personalized and effective treatment options.

## 1 Introduction

Immunotherapy refers to a class of treatments that recognize and attack tumor cells by activating and enhancing the body’s own immune system. This method aims to suppress and eliminate tumors through various strategies, including immune checkpoint inhibitors (ICIs), cytokine therapy, cell therapy, therapeutic vaccines, and integrated immunotherapy strategies. ICIs, targeting programmed cell death protein 1 (PD-1), programmed cell death protein 1 ligand (PD-L1), or cytotoxic T-cell antigen 4 protein (CTLA-4), have proven to be effective for many patients with solid tumors, especially those with advanced-stage melanoma, renal cell carcinoma (RCC), and non–small cell lung cancer (NSCLC) (1–4). Despite their successes, the overall effectiveness of tumor immunotherapy remains relatively low, with at least 50% of patients experiencing primary or acquired drug resistance to treatment, resulting in no therapeutic benefit (4, 5). This challenge is partly due to the absence of precise biomarkers that can accurately identify individuals likely to respond to treatment, as well as those who may not benefit or could experience hyperprogression. Although PD-L1, tumor mutational burden (TMB), and microsatellite instability-high are clinically used to predict the efficacy of ICIs, those biomarkers have notable limitations (6, 7).

The intestine, the largest immune organ in the human body, contains approximately 1×10^13^-1×10^15^ microorganisms, including bacteria, fungi, viruses, and other microbes (8). These intestinal microbiotas play a crucial role in maintaining gut homeostasis and overall health by participating in a wide range of physiological functions with both local and systemic effects (9). Locally, the microbiota help maintain intestinal barrier integrity and regulate mucosal immunity. Systemically, they influence metabolism, inflammation, hematopoiesis, and immunity regulation (10–12). Dysbiosis, often caused by broad-spectrum antibiotics or chemotherapies, can make the gut vulnerable to pathogenic taxa and reduce the production of crucial microbiota-derived metabolites for immune cell development and maintenance (13). This imbalance is associated with the development of various diseases, including hypertension, diabetes, constipation, diarrhea, colitis, allergic diseases, rheumatic diseases, urinary tract infections, skin aging, acne, osteoporosis, chronic gastritis, liver cirrhosis, and cancers. The imbalance of intestinal microbiota not only participates in the occurrence of gastrointestinal tumors (such as gastric cancer, colorectal cancer (CRC), and gallbladder cancer) but also affects the occurrence and growth of non-gastrointestinal cancers (such as lymphoma, hepatocellular carcinoma, breast cancer, pancreatic cancer, prostate cancer (PCa), sarcoma, and ovarian cancer) (14). The gut microbiota composition can affect the efficacy of ICI therapy in patients with cancer, indicating its potential as a predictive biomarker for immunotherapy effectiveness (15–17). The complex interaction between gut microbiota and the host immune system also suggests a therapeutic role in modulating immunological responses to ICIs.

This review explores the progress in research on the predictive role of gut commensal microorganisms in antitumor immunotherapy, particularly ICIs. It also assesses current evidence that modulation of the gut microbiota can enhance ICIs outcomes in patients and highlight promising strategies that could open new avenues for cancer immunotherapy. Additionally, this review discusses the challenges of translating gut microbial biomarkers into clinical practice and developing gut microbiota intervention strategies for immunotherapy.

## 2 Gut microbiota as biomarker for the prediction of immunotherapy efficacy

A growing number of studies have found associations between gut microbes and cancers such as gastrointestinal tumors, melanoma, lung cancer, urogenital and reproductive tumors (**Table 1**). Specific microbial communities and strains may influence cancer risk and development. Microbial communities can influence the activity of the host immune system and have an impact on immune surveillance and treatment of cancer.

**Table 1:** The role of intestinal microecology in predicting the efficacy of immunotherapy.

Tumor	Cancer types	Disease stage	Immunotherapy strategies	Sample types	Sample size	Detecting techniques	References
Melanoma	melanoma	metastatic	CTLA-4 blockade	gut microbiota	25	16S rRNA	(37)
	melanoma	metastatic	CTLA-4 blockade, ipilimumab	gut microbiota	34	16S rRNA	(41)
	melanoma	metastatic	CTLA-4 blockade, ipilimumab	gut microbiota	26	16S rRNA	(39)
	melanoma	Stage III-IV	anti-PD-1	oral and gut microbiota	43	16S rRNA	(17)
	melanoma	metastatic	anti-PD-1	gut microbiota	42	16S rRNA/metagenomic shotgun sequencing	(15)
	melanoma	metastatic	CTLA-4/PD-1 blockade	gut microbiota	27	16SrRNA and shotgun metagenome sequencing	(168)
	melanoma	metastatic	CTLA-4 blockade	gut microbiota	50	16S rRNA	(40)
	melanoma	metastatic	PD-1 and CTLA-4	gut microbiota	115	16S rRNA/metagenomic shotgun sequencing	(139)
	melanoma	Stage IV	ICIs	gut microbiota	165	shotgun metagenomic sequencing	(116)
Gastrointestinal cancers	GI cancer	advanced	PD-1/PD-L1	gut microbiota	74	16S rRNA	(21)
	CRC	mice	ICIs	gut microbiota	/	16S rRNA	(106)
	CRC	metastatic	regorafenib plus toripalimab	gut microbiota	42	16S rRNA	(169)
	CRC	/	PD-L1	gut microbiota	41	/	(24)
	CRC	metastatic	cetuximab + avelumab	gut microbiota	16	16S rRNA	(23)
	CRC	metastatic	PD-1	gut microbiota	/	/	(25)
	HCC	Stage C (BCLC)	anti–PD-1	gut microbiota	8	Metagenome sequencing	(32)
	GC	advanced	ICIs	gut microbiota	77	13C-urea breath test (13C-UBT), H. pylori facal antigen (HpSA) test and histopathology	(170)
	GCs	advanced/unresectable	ICIs	gut microbiota	95	Metagenome sequencing	(171)
	HER2-negative GC	/	Anti-PD-1/PD-L1 immunotherapy or combination therapy	gut microbiota	117	mNGS	(28)
	GC	advanced	/	/	215	breath test, fecal antigen test, histopathology, and/or chart documentation	(30)
	ESCC	human + mice	neoadjuvant chemoimmunotherapy	gut microbiota	40	/	(31)
Lung cancer	NSCLC	advanced	nivolumab	gut microbiota	37	16SrRNA	(51)
	NSCLC	advanced	first- or second-line ICIs	gut microbiota	338	shotgun-metagenomics-based microbiome profiling	(52)
	NSCLC	mice	anti-PD-1 immunotherapy	gut microbiota	/	FMT and 16S PacBio SMRT sequencing.	(53)
	NSCLC	metastatic	ICIs	gut microbiota and respiratory microbiota	75	16S rRNA	(54)
	NSCLC	stage IIIB or IV	first- or second-line immunotherapy or chemoimmunotherapy	gut microbiota	47	16S rRNA	(55)
	lung cancer	mice	/	/	/	16S rRNA	(172)
	lung cancer	I/II/III/IV	anti-PD-1 immunotherapy	respiratory tract microbiome	84	16S rRNA	(59)
Urogenital neoplasms	BC	early stage	PD-L1	urogenital microbiota	28	16S rRNA	(69)
	PC	middle and advanced	pembrolizumab	gut microbiota	23	16S rRNA	(74)
	RRC	advanced	immune checkpoint blockade	gut microbiota	69	whole genome sequencing	(173)
	RRC	metastatic	nivolumab or nivolumab plus ipilimumab therapy	gut microbiota	31	metagenomic sequencing	(65)
	gynecologic cancer	recurrent endometrial, cervical and ovarian cancer	ICI	/	101	/	(81)

BC, bladder cancer; CRC, colorectal cancer; CTLA-4, cytotoxic T-cell antigen 4 protein; ESCC, esophageal squamous cell carcinoma; ICI, immune checkpoint inhibitor; GC, gastric cancer; NSCLC, non-small cell lung cancer; PC, prostate cancer; RRC, renal cell carcinoma.

### 2.1 Gastrointestinal tumor

Globally, gastrointestinal tumors account for more than 25% of all tumor incidences and approximately 35% of tumor-related deaths (18). Factors such as genetics, environmental risks, smoking, and excessive alcohol consumption have been linked to an increased incidence of these tumors. Specific bacteria and imbalances in the gastrointestinal tract’s bacterial population can lead to the development of gastrointestinal tumors. This occurs through mechanisms such as DNA damage, activation of cancer-promoting signaling pathways, production of harmful metabolites (such as secondary bile acids), and suppression of antitumor immunity (19). Moreover, the gut microbiota may also be related to the effectiveness of cancer immunotherapy (20).

Given the crucial role of gut microbiota in the immune response to ICIs, numerous studies have explored the connection between the characteristics of gut microbes and the outcomes of CRC immunotherapy and survival rates. Peng et al. conducted a study on 74 patients with advanced gastrointestinal cancer who were undergoing anti-PD-1-to-PD-L1 immunotherapy. Analysis of their baseline fecal samples showed that an increased Prevotella/Bacteroides ratio was associated with a better response to the treatment (21). A phase Ib/II clinical trial on the combination of regorafenib and toripalimab in treating metastatic CRC found that non-responders had a higher prevalence of *Fusobacterium* compared with responders (22). Additionally, in a group of patients with CRC receiving cetuximab and avelumab, those with longer periods of progression-free survival (PFS) had higher levels of butyrate-producing bacteria *Agathobacter* M104/1 and *Blautia* SR1/5 in fecal samples from five long-term responding patients compared with nine patients with shorter PFS (23). Conversely, Gao et al. discovered that higher levels of *F. nucleatum* were linked to better responses and longer PFS to PD-1 blockade in patients with CRC, which through recruiting IFNc+ CD8+ tumor-infiltrating lymphocytes (TILs) (24). However, Jiang et al. observed that patients with metastatic CRC involving poor responses to immunotherapy had higher levels of *F. nucleatum* and succinic acid, suggesting a mechanism that *F. nucleatum*–derived succinate inhibits the cGAS-interferon-β pathway, thereby suppressing the antitumor response by limiting the *in vivo* CD8+ T-cell transport to the tumor microenvironment (TME). Treatment with metronidazole decreased *F. nucleatum* abundance, which, in turn, lowered succinic acid levels and improved sensitivity to immunotherapy (25). Gao et al. found that Lactobacillus rhamnose-ProBIO-M9 can regulate and improve the diversity and microbial composition of the flora, improve the synthesis of α-ketoglutaric acid, a key metabolite of intestinal flora and host immunity, and thus enhance the anti-CRC tumor immune response (26). Roberti et al. also demonstrated through mouse experiments that oxaliplatin induced immunogenic cell death is mediated by T follicular helper cell (TFH) immune response, which is completely absent in germ-free mice and TLR2/4 knockout mice (27).

Han et al. studied 117 human epidermal growth factor receptor 2 (HER2)-negative patients with advanced gastric cancer who underwent treatments such as chemotherapy alone, immunotherapy alone, and immune-combination chemotherapy regimens. Their research revealed a significant enrichment of lactobacilli in the baseline samples of patients who showed a better response to PD-1/PD-L1 immunotherapy. Moreover, a significant positive correlation with PFS benefit was observed (28). The DELIVE clinical trial aimed to determine the effect of gut microbiome composition on the efficacy of nivolumab in treating advanced gastric cancer. The findings indicated that *Odoribacter* presence was linked to progressive gastric cancer, whereas *Veillonella* presence was associated with either disease remission or disease stabilization, suggesting its potential as a specific biomarker for advanced gastric cancer (29). Furthermore, in patients with metastatic gastric cancer treated with ICIs, those with *H. pylori* infection had significantly shorter median PFS (3.2 vs 6.8 months, hazard ratio [HR] 1.96, p<0.01) and median overall survival (9.8 vs 17.9 months, HR 1.54, p=0.02). This confirmed *H. pylori* infection as an independent predictor of both PFS and OS (30).

Wu et al. discovered that in resectable esophageal squamous cell carcinoma, variations in intratumoral microbiota signatures (specifically β-diversity) predicted the effectiveness of neoadjuvant immune-combination chemotherapy. A positive correlation was found between *Streptococcus* enrichment, Granzyme B+ (GrzB+) and CD8+ T-cell infiltration in tumor tissue, and prolonged disease-free survival (31). In a study involving eight patients with hepatocellular carcinoma treated with PD-1 inhibitors, fecal samples were analyzed, revealing that the three responders had higher microbial richness and more gene counts than those of five non-responders (32).

These studies suggest that gut microbial characteristics could serve as potential predictive markers for the efficacy of immunotherapy in gastrointestinal tumors, indicating a significant link between the intestinal microbiome and immunotherapy effectiveness. In the future, fecal microbiota transplantation (FMT) and other methods may be utilized to influence the success of antitumor immunotherapy. Although existing studies indicate a significant association between gut microbiota characteristics and immunotherapy efficacy, several limitations persist. Firstly, the small sample sizes, primarily focused on colorectal, gastric, and esophageal cancers, may not adequately represent the microbial characteristics of various gastrointestinal tumors. Secondly, while the influence of different microorganisms on immune responses has been explored, the specific mechanisms remain insufficiently studied. For instance, *F. nucleatum* has demonstrated inconsistent results across studies, highlighting the need for further investigation into its mechanisms. Additionally, the heterogeneity in study designs and analysis methods affects the comparability of findings, and many studies overlook patient drug histories and comorbidities. The absence of long-term follow-up data also restricts our understanding of how microbiome changes impact prognosis. Finally, the lack of consistent and standardized biomarkers to predict immunotherapy responses hinders the clinical application of these findings.

### 2.2 Melanoma

Melanoma is associated with an annual mortality rate exceeding 3.5%, with only 15%-20% of patients with metastatic melanoma (MM) (33) surviving past 5 years. The current immune standard of care for patients with MM includes monoclonal antibodies (such as nivolumab and pembrolizumab) targeting PD-1 and PD-L1 (34), as well as therapies targeting the CTLA-4 (such as ipilimumab) (35). Preclinical mouse models have identified several potentially beneficial microbes, including *Bacteroides fragilis*, *Bacteroides thetaiotaomicron*, *Bifidobacterium species*, *Faecalibacterium* species, and *Akkermansia muciniphila*, in the context of ICI therapy (36–38). In patients with MM, immunotherapy response has been correlated with specific intestinal flora, and patients who responded well to immunotherapy had different intestinal flora compared with those who responded poorly compositions (15, 17, 39).

*Faecalibacterium prausnitzii*, in particular, has been linked to improved therapeutic outcomes in CTLA-4 inhibitor-treated patients with MM. A study involving 26 patients with MM receiving ipilimumab showed that those with baseline microbiota enriched in *F. prausnitzii* and other Firmicutes had better outcomes (39). Similarly, high baseline abundance of *Faecalibacterium* was associated with longer PFS in patients treated with CTLA-4 inhibitors (40). However, ipilimumab-induced colitis was more frequent in patients with a higher presence of Bacteroidetes bacteria (41).

The Ruminococcaceae family has been associated with stronger antitumor immune responses in patients treated with PD-1/L1 inhibitors. Gopalakrishnan et al. found that responders to PD-1 inhibitors had higher alpha diversity and relative abundance of Ruminococcaceae bacteria, correlating with enhanced antitumor immune responses (17). Another study on patients with anti-PD-1-treated melanoma revealed that bacteria linked to favorable responses belonged primarily to the Actinobacteria phylum and the Lachnospiraceae/Ruminococcaceae families of Firmicutes. A time-to-event analysis indicated that the composition of baseline microbiota was optimally associated with clinical outcomes approximately 1 year after treatment initiation (42). In responders to PD-L1 inhibitors, baseline fecal samples contained a richer diversity of bacterial species, including *Bifidobacterium longum*, *Collinsella aerofaciens*, and *Enterococcus faecium*. Transplantation of these fecal samples into germ-free mice resulted in an enhanced antitumor effect of the immune response to PD-1/PD-L1 inhibitors (15).

The studies indicate that commensal microorganisms can predict the effectiveness of cancer checkpoint immunotherapy. Preclinical investigations have explored the impact of vancomycin-induced changes in gut flora on the efficacy of chimeric antigen receptor (CAR) T-cell immunotherapy. In a CD19-B16 melanoma mouse model, mice receiving both vancomycin and CD19-targeted CAR T-cells (CART-19) demonstrated superior tumor control and increased tumor-associated antigen (TAA) cross-presentation than those treated with CART-19 alone. This suggests that altering the gut microbiota with vancomycin could enhance outcomes for various tumor types following CAR T-cell therapy (43).

Recent studies, however, have shown that the relationship between gut microecology and immunotherapy effectiveness varies by cohort. A meta-analysis involving 130 patients treated with ICIs for melanoma identified differences in microbiome composition between responders and non-responders; for instance, responders (44) had higher levels of *Faecalibacterium*. Simpson et al. prospectively analyzed patients with melanoma undergoing neoadjuvant therapy with the combination of ipilimumab and nivolumab, and the results showed that responders possessed microbiomes dominated by Ruminococcaceae, highlighting that a fiber-influenced microbiome alone does not guarantee a response (44). Additionally, Lee et al., examining patients with advanced cutaneous melanoma treated with ICIs (n = 165), integrated findings with 147 macrogenomic samples from previous studies, identifying a link between gut microbiome composition and ICI response, involving species such as *Bifidobacterium pseudoatenulatum*, *Roseburia* spp., and *Akkermansia muciniphila* (44). However, this association was contingent on cohort-specific correlations, and no single microbial species emerged as a consistently reliable biomarker across different studies.

In conclusion, the microbiome presents a promising pathway for predicting the efficacy of immunotherapy in melanoma. However, the influence of the human gut microbiome on responses to immune checkpoint inhibitors (ICIs) is complex, extending beyond merely identifying the presence or absence of specific microbial species in responders versus non-responders. Additionally, the relationship between certain microorganisms, such as *F. prausnitzii* and *Ruminococcaceae*, and immune responses varies significantly among patients, and there is currently a lack of consistent biomarkers for clinical application. Furthermore, the small sample sizes and limited diversity in existing studies constrain the generalizability of the findings. Future research should prioritize increasing sample sizes, exploring the specific mechanisms underlying microbial immune responses, and fostering the development of personalized treatment strategies.

### 2.3 Lung cancer

Lung cancer remains the leading cause of cancer-related mortality globally, categorized into small cell lung cancer (SCLC) and NSCLC (45) based on pathology, with NSCLC comprising 80%-85% of cases. Despite the availability of treatments such as radiotherapy, targeted therapy, and immunotherapy, the average 5-year survival rate of patients with lung cancer is only 20% (46). However, the application of ICIs has progressively improved survival rates for patients with advanced NSCLC by inhibiting tumor cell–expressed tolerance pathways and preserving the immune system’s cancer-suppressing functions. Emerging evidence underscores the gut microbiota’s role in modulating ICI treatment responses, indicating its importance in tumor immune surveillance and efficacy prediction of ICIs (47).

A substantial body of research connects the gut microbiome with the response to ICI therapy in both preclinical and clinical settings. Routy et al. discovered an association between clinical response to ICIs and the relative abundance of *Akkermansia muciniphila*. Zhu et al. identified that butyrate, a gut microbiota-derived metabolite, boosts the effectiveness of anti-PD-1 immunotherapy by affecting cytotoxic CD8 T cell signaling, marking butyrate as a potential biomarker (48). The gut microbiota influences the function of both innate (dendritic cells, macrophages, and natural killer cells) and adaptive (CD8+ T cells, CD4+ T cells) immune cells, thereby modifying the TME and the host’s response to ICIs + T cells). In addition, GM alters TME immunity and host ICI responses (49).

The gut microbiota plays a crucial role not only in regulating the immune response of the gastrointestinal tract but also in influencing the health and diseases of distal organs such as the respiratory system through its microecology (50). A study involving 37 Chinese patients with advanced NSCLC treated with nivolumab, as part of the CheckMate-078 and CheckMate-870 trials, revealed that patients with high gut microbiome diversity experienced longer PFS. The study also noted differences in microbiome composition between those who responded to the treatment and those who did not. Interestingly, prior antibiotic therapy appeared not to influence the outcomes, although it’s worth noting that the sample size was small. High microbiome diversity correlated with enhanced characteristics of memory CD8+ T cells and NK cells, as determined by flow cytometry in peripheral blood (51). Derosa and colleagues explored the link between fecal *Akkermansia muciniphila (Akk)* and the clinical benefits of ICIs in patients with NSCLC, finding baseline levels to be predictive of increased response rates and overall survival, irrespective of PD-L1 expression, antibiotics, and performance status (52). Huang et al. investigated the effects of combining ginseng polysaccharides (GPs) and αPD-1 monoclonal antibody (mAb) on tumor response in mice, using FMT and 16S PacBio single-molecule real-time (SMRT) sequencing. They found that GPs enhanced the antitumor response to αPD-1 mAb by increasing the levels of the microbial metabolite valeric acid and reducing both the ratio of L-kynurenine and the Kyn/Trp ratio. Notably, *Parabacteroides distasonis* and *Bacteroides vulgatus* were more prevalent in those responding to treatment with anti-PD-1 blockers than in non-responders (53). Another study identified different microbial signatures between gut and respiratory microbiota, noting that only the gut microbiota’s alpha diversity correlated with a response to anti-programmed death receptor-1 therapy. A higher alpha diversity in the gut microbiota was associated with better responses and longer PFS (54). Grenda et al. discovered that certain bacterial families Barnesiellaceae, Ruminococcaceae, Tannerellaceae, and Clostridiaceae could influence immunotherapy outcomes (55), with higher abundances linked to extended PFS. High abundance of *Bacteroidaaceae*, *Barnesiellaceae*, and *Tannerellaceae* can prolong PFS. Future research could leverage next-generation sequencing (NGS) to pinpoint bacteria at the species or subspecies level that predict immunotherapy efficacy.

The lung microecology encompasses the specific microorganisms present in the lungs, their genetic information, and their interactions (56). Pathological conditions in lung diseases alter the growth conditions for these microorganisms, leading to a disruption of lung microecology and further pathological processes. This perpetuating cycle is a critical factor in the development and progression of lung diseases (57). Despite the focus on the gut microbiome in most lung cancer microbiome studies, evidence from preclinical mouse studies indicates that the lower respiratory microbiome has a significant impact on local immunity and could be a more accurate predictor of immunotherapy outcomes in lung cancer than the gut microbiome (58). Tsay et al. discovered that patients with stage IIIB-IV lung cancer involving lymph node metastasis commonly exhibit lower airway dysbiosis, which correlates with poor prognosis. This dysbiosis is linked to an upregulation of the IL17, PI3K, MAPK, and ERK pathways, primarily driven by *Veillonella parvula*. In a KP lung cancer mouse model, *V. parvula*-induced lower airway dysbiosis led to decreased survival, increased tumor burden, an IL17 inflammatory phenotype, and activation of checkpoint inhibitor markers (58). Jang et al. found distinctions in microbiota composition related to PD-L1 expression levels and immunotherapy response, with *Veillonella dispar* more prevalent in patients with high PD-L1 levels (≥10%), and *Neisseria* in those with low PD-L1 levels (<10%). The presence of *V. dispar* was dominant in the immunotherapy responder group, whereas *Haemophilus influenzae* and *Neisseria perflava* were more common in the non-responder group. The findings suggest that the abundance of *Neisseria* and *V. dispar* correlates with PD-L1 expression and immunotherapy (59) responses. Additionally, modulating lung microbiota with aerosolized antibiotics has shown promise in enhancing immunity against lung metastases in patients with melanoma (60). Research has primarily utilized metagenomic shotgun sequencing, 16S rRNA gene sequencing, and quantitative polymerase chain reaction techniques to explore the diversity and abundance of the bacterial microbiome in fecal or respiratory tract samples (61).

The gut and lung microbiomes may serve as important biomarkers for predicting immunotherapy responses, and their modulation could enhance reactions to immune checkpoint inhibitors (ICIs). Further research is essential for understanding the cancer-microbial-immune axis and its relationship with host immunity, positioning the microbiome as a valuable clinical predictive tool. Despite significant advancements in studying the gut microbiota’s influence on lung cancer immunotherapy, particularly regarding treatment resistance and efficacy prediction, limitations remain. Current studies often have small, non-diverse samples, making universal conclusions challenging. Moreover, the specific mechanisms of microbial immune responses, particularly those involving *F. nucleatum* and *A. muciniphila*, have not been fully explored. Increasing evidence suggests that the lung microbiome may play a crucial role in immunotherapy outcomes. Future studies should therefore broaden sample sizes and utilize advanced sequencing techniques to investigate interactions between gut and lung microbiomes, aiming to enhance strategies for lung cancer immunotherapy.

### 2.4 Urinary system tumor

Urologic tumors, which include cancers of the kidney, ureter, bladder, and urethra, present significant health challenges. RCC is a primary form of kidney cancer that often remains asymptomatic until reaching advanced stages (62). Approximately one-third of patients will progress to a stage that is either locally advanced or metastatic, with recurrences and distant metastases common following nephrectomy (63). Despite recent advancements in treatment, options are still limited and often ineffective. Risk factors for RCC include obesity, hypertension, smoking, and chronic kidney disease (64). Recent research highlights the microbiome’s potential role in promoting RCC, with studies showing how manipulating the microbiota might enhance the effectiveness of RCC treatments and predict treatment outcomes. Salgia et al.’s research on the gut microbiome of patients with RCC undergoing immunotherapy found a link between higher microbial diversity and improved treatment results, indicating the microbiome’s potential to enhance cancer immunotherapy outcomes. Treatment response is influenced by changes in microbial species during treatment. These changes in microbiome composition over time indicate the potential for improving cancer immunotherapy outcomes by altering the microbiome (65).

Bladder cancer is among the most prevalent cancers worldwide, affecting nearly 500,000 people annually, with more than a third succumbing to the disease (66). The causes of bladder cancer are complex and not fully understood, involving factors such as smoking, occupational exposure to certain chemicals, specific medications, and a history of radiotherapy (67). Recently, the role of the human microbiome in the development of chronic diseases, including bladder cancer, has gained attention (68).

The relationship between urogenital microbiota and bladder cancer has garnered significant interest. A particular study examined how urogenital microbial communities correlate with PD-L1 expression in male patients with non–muscle-invasive bladder cancer (NMIBC). Based on PD-L1 immunohistochemistry results, participants were categorized into a PD-L1-positive group (Group P) and a PD-L1-negative group (Group N). Group P demonstrated a higher species richness. Further analysis indicated that an increase in PD-L1-positive cells was associated with enhanced richness of the urogenital microbiota. Notable differences in the urogenital microbiota’s composition were observed between Groups P and N. Specifically, Group P showed an enrichment of certain bacterial genera (e.g., *Leptotrichia*, *Roseomonas*, and *Propionibacterium*) and a decrease in others (e.g., *Prevotella* and *Massilia*) compared to Group N (69).

PCa is the most prevalent non-skin cancer among men and is a leading cause of cancer-related deaths globally (70). PCa is a complex, multifactorial disease influenced by genetic, environmental, and physiological factors. Risk factors for PCa include family history, age, diet, ethnicity, and viral and bacterial infections (71). Despite the availability of conventional treatments such as prostatectomy, chemotherapy, radiotherapy, and androgen deprivation therapy (ADT) that can improve survival rates for patients with metastatic PCa, the 5-year survival rate remains approximately 30% (72). The potential direct or indirect links between cancer, including PCa, and specific microbiota have been the focus of extensive research in recent years (73).

A study by Peiffer et al. explored the effect of urogenital microbiota on the response to immunotherapy in advanced metastatic castrate-resistant PCa (mCRPC). The research aimed to profile the microbiome composition of 23 mCRPC patients treated with enzalutamide and pembrolizumab. The study assessed microbial diversity in fecal samples before and after pembrolizumab treatment the reported composite index associated with checkpoint inhibitor response. The results showed little difference in α and β diversity between responders and non-responders. However, responders to pembrolizumab treatment had an increase in the oral bacterium *Streptococcus salivata* and a decrease in the intestinal bacterium *Akkermansia* (74).

The microbiome of urinary system tumors (such as renal cell carcinoma, bladder cancer, and prostate cancer) may influence immunotherapy, but several differences exist. Renal cell carcinoma often presents asymptomatically, leading to advanced diagnoses and limited early treatment options. While the microbiome’s role in RCC treatment is noted, studies typically involve small sample sizes and lack long-term tracking of changes. In bladder cancer, the urinary microbiota is linked to PD-L1 expression, yet the mechanisms remain unclear. Prostate cancer research shows minimal microbiota differences in response to immunotherapy, limiting biomarker potential. Future studies should increase sample sizes and explore the microbiome’s mechanisms in cancer progression and treatment response to enable personalized therapies and improve patient outcomes.

### 2.5 Reproductive system tumors

Gynecologic malignancies, including cancers of the uterus, cervix, ovary, vulva, and vagina, pose a significant health burden (75). These cancers have a complex and multifactorial etiology, involving diverse genetic, epigenetic, immunologic, and environmental risk factors (76). Recently, the microbiome has emerged as a notable environmental risk factor for cancer, including gynecologic malignancies (77) these cancers. Various studies indicate that certain bacteria or microbial communities could play a role in the onset of gynecologic cancers. Moreover, the microbiota can affect the toxicity and effectiveness of treatments such as chemotherapy, immunotherapy, and radiation therapy in women with these gynecologic malignancies (78).

The diversity and composition of the endometrial microbiota are crucial in the immune pathogenesis of endometrial cancer. Evidence is mounting that disruptions in the reproductive tract’s microbiome, specific bacteria, and cytokines may actively contribute to the development or progression of HPV infection, cervical intraepithelial neoplasia, and cervical cancer (79). Li et al. showed that in a mouse model of ovarian cancer, the vaginal microbiota was disrupted with altered metabolite profiles, potentially due to changes in amino acid or lysophosphatidylcholine metabolism. Broad-spectrum antibiotics have proven effective in reversing microbiota dysregulation and inhibiting carcinogenic progression. Moreover, certain vaginal bacteria such as *Burkholderia* have been studied as non-invasive biomarkers (80). A retrospective cohort study involving 101 women with recurrent endometrial, cervical, and ovarian cancer indicated that antibiotic treatment before starting immunotherapy significantly reduced treatment response, time to progression, and survival (81). Understanding the microbiome’s role in modulating gynecologic tumors’ response to immunotherapy could significantly enhance therapeutic outcomes. Future research should focus on leveraging gut microbiota to predict immunotherapy efficacy for reproductive system tumors.

In addition to bacteria, fungi within the gut microbiome also exhibit immunosuppressive and carcinogenic effects (82). A recent study analyzed fecal metagenomes from cancer patients undergoing ICI treatment to identify fungi with differential abundance. These could serve as biomarkers for predicting responses to ICI treatment. The findings indicated that intestinal fungi (area under the curve [AUC]=0.87) were more predictive than bacteria (AUC=0.83), and combining both fungi and bacteria yielded the highest predictive accuracy (AUC=0.89). Furthermore, it was found that *Schizosaccharomyces octosporus*, in responders, ferments starch into short-chain fatty acids, which are known to have protective effects against cancer (83, 84). This study is the first to demonstrate the potential of fungi in predicting the efficacy of immunotherapy efficacy and enhancing response rates, offering novel insights into predicting immune checkpoint blockade therapy’s effectiveness (85).

Although more and more research is showing the importance of the microbiome in female reproductive system tumors (such as uterine, cervical, ovarian, etc.), there are still some limitations. First of all, most of the existing studies are small-scale or animal model experiments, lacking the data support of large-scale clinical trials. As a result, the microbiome and its complex relationship to cancer development is not yet fully understood. In addition, although specific bacteria and fungi may serve as biomarkers, their specific mechanisms remain unclear, especially their role in anti-tumor therapy. Future research should focus on uncovering the specific role of the microbiome in tumorgenesis and its response to immunotherapy, particularly through large-scale clinical trials to confirm the potential of the microbiome as a biomarker. In addition, exploring the relationship between the gut microbiome and immunotherapy efficacy will facilitate the development of personalized treatment strategies to improve patient outcomes.

## 3 Manipulation of the microbiota to modulate immunotherapy in cancer

Demographic factors such as age and gender) and environmental factors (such as diet, geographic location, and lifestyle), rather than genetics, primarily determine the composition of the gut microbiome in healthy individuals (86). Patients with cancer often undergo treatments such as chemotherapy and radiation before immunotherapy, which can alter the gut microbiota, thereby affecting the efficacy of immunotherapy. The gut microbiome profoundly influences the clinical responses and outcomes of patients receiving cancer immunotherapy (87). Thus, manipulating gut microbiota composition to a status of optimal biodiversity and signature before immunotherapy might be an effective approach to improve the efficacy of immunotherapy. Altering the gut microbiota is expected as a novel method to deal with resistance and improved diseases associated with intestinal dysbiosis. Potential routes to target gut microbiota community include FMT, probiotics, prebiotics, synbiotics, postbiotics, and diet.

### 3.1 Fecal microbiota transplantation

Recently, FMT has garnered substantial interest for its potential to improve outcomes in cancer therapy and address treatment-related complications (88, 89). FMT involves transferring functional gut flora from a healthy donor’s fecal into a patient’s intestinal tract to rebalance the gut flora and treat intestinal and extraintestinal diseases (90, 91). Considered a groundbreaking method for its ability to modify the gut microbiota (88), FMT’s history dates back over 1,000 years to the Chinese “yellow dragon soup” for intestinal issues. The process includes homogenizing and filtering donor fecal for transplantation through methods such as colonoscopy, enema, or other means. Common delivery routes for FMT include upper gastrointestinal routes (e.g., nasogastric/nasojejunal tube, endoscopy, oral capsules) and lower gastrointestinal routes (e.g., retention enema, sigmoidoscopy, or colonoscopy) (92). FMT has shown remarkable efficacy against *Clostridium difficile* infection, with a 90% cure rate, making it highly recommended for treating recurrent or refractory *Clostridium difficile* infection (93). Over the past decade (94), FMT has been applied to treat more than 85 diseases globally. Considering the crucial link between the gut microbiota and the immune system, and the predictive value of gut microbiota for immunotherapy effects, numerous studies have explored FMT as a means to enhance the efficacy of antitumor immunotherapy (95).

#### 3.1.1 Pre-clinical studies

Compared with mice receiving FMT from melanoma patients who did not respond to anti-PD-1 therapy, those that received FMT from responders showed increased infiltration of CD8+ T-cells within tumors and an enhanced effectiveness of anti-PD-1 therapy. Preclinical mouse models indicate that the gut microbiome influences tumor response to checkpoint blockade immunotherapy. Immune profiling showed improved systemic and antitumor immunity in germ-free mice receiving fecal transplants from melanoma patients who responded well to ICIs (17, 95). Bacterial species more prevalent in responders included *Bifidobacterium longum*, *Collinsella aerofaciens*, and *Enterococcus faecium*. Reconstitution of germ-free mice with fecal material from responders among patients with melanoma led to better tumor control, stronger T cell responses, and increased efficacy of anti–PD-L1 therapy (15). These findings establish a direct link between the gut microbiota and the response to ICIs. Similarly, metagenomic analysis of fecal samples from patients with NSCLC, RCC, and urothelial carcinoma at diagnosis showed a correlation between the clinical responses to ICIs and the relative abundance of *Akkermansia muciniphila*. Oral supplementation of the bacteria with *A. muciniphila* to antibiotic-treated mice after receiving FMT from non-responders restored the effectiveness of PD-1 blockade in an interleukin-12-dependent manner, enhancing the recruitment of CCR9+CXCR3+CD4+T lymphocytes to the tumor beds (12, 16). These studies underscore the significant role of the gut microbiota in the response to cancer immunotherapy and confirm its influence on antitumor immune responses during ICI treatment.

#### 3.1.2 Clinical trials

Numerous patient-centered intervention studies have explored the effects of FMT, employing diverse donor sources and strategies, on the efficacy of ICIs in patients with melanoma and other solid tumor types such as NSCLC, RCC, CRC, and head and neck cancer, in both treatment-naive and refractory situations. These investigations into the safety, feasibility, and effectiveness of FMT for cancer patients not responding to ICI therapy, with donors being ICI responders, have led to several ongoing clinical trials (**Table 2**). Two significant single-arm, open-label clinical trials aimed to evaluate the safety, feasibility, and effect on immune cells of FMT and re-induction of anti-PD-1 immunotherapy in patients with refractory MM. The phase I trial by Baruch et al. showed that out of 10 patients with anti-PD-1 therapy-resistant malignant melanoma who underwent FMT, one achieved complete remission and two partial remission. In these three responders, increased immune activity (CD8+ T cells) was observed in both the gut mucosa and the TME (NCT03353402) (96). Another phase II study reported that 6 out of 15 patients resistant to PD-1 inhibitors experienced clinical benefits after receiving FMT from PD-1 inhibitor responders, including increased tumor microbial diversity, activation of CD8+ T cells, and a reduction in IL-8+ myeloid cells(NCT03341143) (97). These early results are encouraging, suggesting that FMT and anti-PD-1 therapy can alter the gut microbiome and reprogram the TME to overcome ICI therapy resistance in some advanced melanoma cases (12). Ongoing clinical trials are examining the safety, feasibility, and effectiveness of FMT in patients with other solid tumor types, with many being multicenter randomized controlled trials (RCTs) involving a larger number of participants (**Table 2**). Further extensive studies are required to assess its effectiveness.

**Table 2:** Clinical trials on gut microbiome modulation in cancer immunotherapy.

NCT number	Year and Country	Title	Study design	Patients	Sample size	Intervention	Outcome Measures	Reference
NCT03341143	2017, USA	Phase II feasibility study of FMT in advanced melanoma patients not responding to PD-1 blockade	single group assignment, open label, phase II	melanoma patients primarily resistant to PD-1 inhibitor therapy	18	FMT together with pembrolizumab	ORR	(97)
NCT03353402	2017, Israel	Altering the gut microbiota of melanoma patients who failed immunotherapy using FMT from responding patients	single group assignment, open label	melanoma patients who failed immunotherapy	40	FMT from immunotherapy responding patients	incidence of FMT-related AEs; proper implant engraftment	(96)
NCT04758507	2021, Italy	Targeting gut microbiota to improve efficacy of immune checkpoint inhibitors in patients with advanced renal cell carcinoma	randomized, parallel assignment, double-blind	renal cell carcinoma patients received ICIs	50	FMT (from donors who are responding to ICIs) or Placebo FMT	CRR	NA
NCT04130763	2019, China	Investigator-initiated trial of fecal microbiota transplant (FMT) capsule for improving the efficacy of anti-pd-1 in patients with pd-1 resistant digestive system cancers	single group assignment, open label	patients with anti-PD-(L)1 resistant/refractory digestive (including gullet, stomach and intestine) system cancers	10	FMT capsule combined with anti-PD-1 therapy	ORR, rate of AEs	NA
NCT04924374	2021, Spain	Microbiota transplant in advanced lung cancer treated with immunotherapy	randomized, parallel assignment, open label	patients with advanced lung cancer who received immunotherapy	20	fecal microbiota capsules from healthy donors or long-term survivors of advanced lung cancer	safety	NA
NCT04729322	2021, USA	Pilot trial of fecal microbiota transplantation and re-introduction of anti-pd-1 therapy in dMMR colorectal adenocarcinoma anti-PD-1 non-responders	Non-randomized, parallel assignment, open label, phase II trial	CRC patients who didn’t respond to anti-PD-1 therapy	15	fecal microbiota capsules from PD-1 responding dMMR CRC patients to anti-PD-1 therapy	ORR	NA
NCT05286294	2017, Norway	MITRIC: microbiota transplant to cancer patients who have failed immunotherapy using fecal from clinical responders	single group assignment, open label, phase IIa	patients who didn’t respond to ICI therapy	20	FMT from ICI-responders	AEs, ORR;	NA
NCT03772899	2017, USA	Fecal microbial transplantation in combination with immunotherapy in melanoma patients (mimic)	single group assignment, open label, phase I	advanced melanoma who received ICIs	20	receive FMT at least one week prior to treatment with ICIs	safety	NA
NCT04951583	2021, Canada	Phase II trial of fecal microbial transplantation in patients with advanced non-small cell lung cancer and melanoma treated with immune checkpoint inhibitors	multi-center single-arm, open-label, phase II trial	patients with metastatic or unresectable non-small NSCLC and melanoma treated with ICIs	70	treated with ICIs combined with investigational FMT capsules	ORR	NA
NCT04577729	terminated, Austria	Inducing remission in melanoma patients with checkpoint inhibitor therapy using fecal microbiota transplantation	randomized, parallel assignment, double-blind	patients experienced disease progression or recurrence during treatment with an anti-PD-1 monoclonal antibody	5	receive fecal from prior MM patients in remission for at least 1 year after ICI treatment	PFS	NA
NCT05251389	2022, Netherlands	Conversion of unresponsiveness to immunotherapy by fecal microbiota transplantation in patients with metastatic melanoma: a randomized phase Ib/IIa trial	randomized controlled trial double-blind	ICI refractory metastatic melanoma patients	24	FMT from an ICI non-responding or responding donor in combination with ICI	clinical benefit	NA
NCT04758507	2021, Italy	Targeting gut microbiota to improve efficacy of immune checkpoint inhibitors in patients with advanced renal cell carcinoma	randomized controlled trial double-blind	patients with advanced renal cell carcinoma who received ICIs	50	Patients will receive donor FMT or placebo FMT	ORR	NA
NCT04988841	2021, France	Prospective randomized clinical trial assessing the tolerance and clinical benefit of fecal transplantation in patients with melanoma treated with CTLA-4 and PD1 inhibitors	multi-center, randomized controlled trial double-blind	patients with melanoma who received immunotherapy	60	Fecal microbiotherapy maat013 or Placebo	AEs	NA
NCT04521075	2021, Israel	A phase Ib trial to evaluate the safety and efficacy of fecal microbial transplantation (FMT) in combination with nivolumab in subjects with metastatic or inoperable melanoma, Microsatellite Instability-high (MSI-H) or mismatch-repair deficient (dMMR) cancer, or Non-Small Cell Lung Cancer (NSCLC)	single group assignment, open label, phase I	patients with metastatic or inoperable melanoma, MSI-H or dMMR cancer, or NSCLC	42	Anti-PD1 plus FMT by capsules	AEs	NA
NCT04116775	2021, USA	A phase II single arm study of fecal microbiota transplant (FMT) in men with metastatic castration resistant prostate cancer whose cancer has not responded to enzalutamide + pembrolizumab	single arm, open-label, phase II	metastatic castration resistant prostate cancer who does not respond to pembrolizumab and enzalutamide	32	FMT from participants who respond to pembrolizumab	percentage of participants with a PSA decline of ≥ 50%	NA
NCT05008861	2021, China	Safety of gut microbiota reconstruction plus PD-1/PD-L1 monoclonal antibodies to treat locally advanced or metastatic non-small cell lung cancer	single arm, open-label	patients with locally advanced or metastatic NSCLC after first-line treatment with PD-1/PDL-1 antibody	20	FMT with anti-PD-1/PD-L1 treatment, capsules contained washed fecal microbiota	FMT-related AEs, anti-PD-1/PD-L1-related AEs	NA
NCT05750030	2023, Austria	Fecal microbiota transplant (FMT) combined with atezolizumab plus bevacizumab in patients with hepatocellular carcinoma who failed to respond to prior immunotherapy - the FAB-HCC pilot study	single arm, open-label	patients with HCC who failed to achieve a complete or partial response to atezolizumab plus bevacizumab	12	FMT from patients with HCC who responded to PD-(L)1-based immunotherapy	treatment-related AEs	NA
NCT04909034	2021, Taiwan	Safety and potential efficacy of MS-20 in combination with pembrolizumab for the treatment of NSCLC	RCT	NSCLC patients who received pembrolizumab	30	fermented soybean extract MS-20 or Placebo	the incidence of AEs	NA
NCT04552418	2020, USA	Pilot study of intestinal microbiome modification with resistant starch in patients treated with dual immune checkpoint inhibitors	single group assignment, open label, early phase I	patients with solid tumor undergoing dual ICIs	12	potato starch (Resistant starch), dietary supplement	AEs of ICI therapy, unanticipated AEs	NA
NCT05083416	2021, USA	Effect of Prolonged nightly fasting (PNF) on immunotherapy treatment outcomes in patients with advanced head and neck cancer (HNSCC)-role of gut microbiome	non-randomized, parallel assignment, open label	head and neck cancer patients who received immunotherapy	29	prolonged nightly fasting (eating within an 8-10-hour window during the day)	rates of prolonged nightly fasting compliance	NA
NCT04645680	2020, USA	Diet and immune effects trial: diet- a randomized double blinded dietary intervention study in patients with metastatic melanoma receiving immunotherapy	randomized, parallel assignment, double-blind, phase II	Patients with advanced melanoma who received pembrolizumab/nivolumab	42	high-fiber diet	change in the gut microbiome	NA
NCT04866810	2021, USA	The effect of diet and exercise on immunotherapy and the microbiome (EDEN)	randomized, parallel assignment, double-blind,	patients with melanoma who will be getting immunotherapy treatment	80	high fiber, exercise	compliance with study requirement (>60%)	NA
NCT05119010	2021, France	A Pilot study evaluating a ketogenic diet concomitant to nivolumab and ipilimumab in patients with metastatic renal cell carcinoma	non-randomized, parallel assignment, open label	patients with metastatic RCC received ipilimumab/nivolumab	60	ketogenic diet (continuous or discontinuous)	ORR	(154)
NCT04208958	2019, USA	Phase I Study of VE800 and nivolumab in patients with selected types of advanced or metastatic cancer	multicenter, single group assignment, open label, phase I	patients with selected types of advanced or metastatic cancer	56	VE800 combined with nivolumab/live biotherapeutic product/oral capsule	AEs, ORR	(105)
NCT03817125	2019, USA	A multicenter phase 1b randomized, placebo-controlled, blinded study to evaluate the safety, tolerability and efficacy of microbiome study intervention administration in combination with anti-PD-1 therapy in adult patients with unresectable or metastatic melanoma	multicenter, randomized, placebo-controlled, phase Ib	participants with anti-PD-1 therapy naïve, unresectable or metastatic melanoma	14	SER-401, or placebo in combination with anti-PD-1 therapy (nivolumab)	AEs	(108)
NCT03686202	2018, Canada	Feasibility study of microbial ecosystem therapeutics (MET-4) to evaluate effects of fecal microbiome in patients on immunotherapy (MET4-ICI)	randomized, single group assignment, open label, phase II	patients with advanced solid tumors already or starting on ICI	65	MET-4 isolated from a fecal sample of a healthy donor/orally	engraftment of MET-4 strains	(107)
NCT03072641	2017, Sweden	Using probiotics to reactivate tumor suppressor genes in colon cancer	randomized, parallel assignment, open label	patients with CRC	20	dietary supplementation consists of two probion clinical tablets	the microbiota composition	(112)
NCT03782428	2018, Malaysia	probiotic effects on clinical and circulating inflammatory cytokines status in patients with colorectal cancer: a randomized double blind clinical trial	randomized double-blind placebo-controlled trial	operable colorectal cancer	52	patients were randomized to receive either placebo or probiotic	level of circulating inflammatory cytokines	(174)
NCT03829111	2019, USA	Pilot study to evaluate the biologic effect of cbm588 in combination with nivolumab/ipilimumab for patients with metastatic renal cell carcinoma	the first randomized, parallel assignment, open label	patients with metastatic renal cell carcinoma who received ICIs (nivolumab plus ipilimumab)	30	live butyrate-producing bacteria (Clostridium butyricum strain CBM588)	the response rate and mPFS	(111)
NA		Association of probiotic Clostridium butyricum therapy with survival and response to immune check- point blockade in patients with lung cancer	retrospectively	advanced NSCLC treated with ICIs	118	probiotic clostridium butyricum therapy was given before and/or after ICI	PFS	(175)
NCT04601402	2020, Germany	A phase I/Ib study to evaluate the safety, tolerability, biological and clinical activities of GEN-001 in combination with avelumab in patients with advanced solid tumors who have progressed during or after treatment with anti-PD-(L)1 Therapy	sequential assignment, open label	locally advanced or metastatic solid tumors who have progressed on at least two lines of approved therapy for their histological subtypes which includes ICI therapy.	11	orally GEN-001	incidence of AEs, laboratory abnormalities, and dose-limiting toxicity	NA
NCT05032014	2021, China	Probiotics enhance the treatment of PD-1 inhibitors in patients with liver cancer	randomized, double-blind, placebo-controlled trial	liver cancer patients who received anti-PD-1 treatment	46	probiotic-M9 isolated from healthy women’s breast milk samples	ORR	NA
NCT05094167	2021, China	The mechanism of probiotic lactobacillus bifidobacterium V9 (Kex02) improving the efficacy of carilizumab combined with platinum in non-small cell lung cancer patients	randomized, double-blind, placebo-controlled trial	NSCLC patients who received PD-1 inhibitor and platinum	46	probiotic-V9 isolated from healthy women’s breast milk sample	ORR	NA
NCT04699721	2020, China	Clinical study of neoadjuvant chemotherapy and immunotherapy combined with probiotics in patients with potential/resectable non-small cell lung cancer	single group assignment, open label	resectable stage III NSCLC patients who received PD-1 inhibitor and chemotherapy	40	Bifico oral taking	AEs, surgical complications, non-R0 surgical events	NA
NCT03358511	2017, USA	Engineering gut microbiome to target breast cancer	single group assignment, open label	operable stage I-III breast adenocarcinoma tumors ≥ 1.0 cm	7	given probiotics prior to surgery	mean number of cytotoxic T lymphocytes	NA
NCT04116658	2019, France	A multicenter, open-label, first-in-human, phase 1b/2a trial of EO2401, a novel multipeptide therapeutic vaccine, with and without check point inhibitor, following standard treatment in patients with progressive glioblastoma	phase 1b/2a, multicenter, non-randomized, sequential, open label	patients with unequivocal evidence of progressive or first recurrent glioblastoma	100	EO2401 is administered alone and in combination with nivolumab, and nivolumab/bevacizumab	safety and tolerability of EO2401	(132, 133)

ORR, objective response rate; CRR, complete response rate; DCR, disease control rate; DOR, duration of response; OS, overall survival; PFS, progression-free survival; NSCLC, non-small cell lung cancer; FMT, fecal microbiota transplant; PBMC, peripheral blood mononuclear cell; MPR, major pathologic response; DFS, disease free survival; RT, recurrence rate; SD, stable disease; PR, partial response; CR, complete response, dMMR, mismatch-repair deficiency; CRC, colorectal cancer.

Beyond modulating responsiveness to ICIs, FMT might also play a role in alleviating immune-related adverse events (irAEs). In 2018, Wang et al. reported two cases where ICI-associated colitis, persistent despite corticosteroid and infliximab treatment, was successfully treated with FMT. This treatment reduced inflammation and healed ulcerations, as confirmed by colonoscopy, due to the reconstitution of the gut microbiome and an increase in regulatory T cells within the colonic mucosa. These initial findings suggest that altering the gut microbiome could counteract ICI-associated colitis (98). A subsequent larger clinical trial (NCT03819296) conducted by the same team enrolled 47 patients with stubborn ICI-associated colitis. Early results showed an 85.1% symptom response rate after FMT, with a median response time of 4.5 days. By the end of the study, 87.2% of patients achieved clinical remission. These outcomes indicate that FMT could be an effective remedy for stubborn ICI-associated colitis, with a low complication rate (99). Another ongoing single-arm trial is exploring FMT as a supportive measure to mitigate toxicity from the ipilimumab and nivolumab combination in patients with RCC (NCT04163289).

However, the safety of FMT remains under-scrutinized. In 2019, researchers from Massachusetts General Hospital reported two cases of extended-spectrum beta-lactamase-producing *Escherichia coli* bacteremia following FMT in separate clinical trials, leading to one patient’s death (100). This resulted in a safety warning from the FDA, emphasizing the life-threatening risk of infection from FMT and the necessity for donor facal screening for antibiotic-resistant bacteria. Additionally, the variability in FMT due to donor differences, and the limitations of crude fecal transplants in consistency and acceptability are noted. The efficacy of FMT can also be influenced by the manufacturing process of fecal preparation, doses, and delivery routes (93). Fortunately, transplantation of more precise and efficacious microbial components may be an effective way to overcome the heterogeneity of crude FMT that only involves manual suspension and filtration steps. The washed microbiota transplantation (WMT) developed by Nanjing Medical University involves automatic repeated centrifugation plus suspension thus viruses and pro-inflammatory could be washed out during the washing process (101). Due to its safety, precision, and quality control, WMT was officially endorsed as a consensus by the FMT-standardization Study Group in 2020 (102, 103).

FMT has shown promise in enhancing cancer therapy outcomes, particularly in managing Clostridium difficile infections and modulating responses to ICIs. However, its implementation faces significant limitations, including safety concerns, variability due to donor differences, and the impact of preparation methods on efficacy. Notably, cases of infection from antibiotic-resistant bacteria post-FMT highlight the need for stringent donor screening. Future research should prioritize enhancing safety protocols, exploring precise microbial transplantation techniques like WMT, and conducting larger multicenter clinical trials to assess FMT’s efficacy across various solid tumors. Additionally, investigating FMT’s potential to alleviate immune-related adverse events will be crucial in optimizing cancer immunotherapy. Understanding the intricate relationship between gut microbiota and the immune system will deepen insights into FMT’s role, paving the way for its broader application in cancer treatment.

### 3.2 Bacterial consortium

An alternative strategy for modulating the gut microbiota involves using a carefully selected consortium of bacterial strains. This approach reduces the risks and variability associated with FMT while preserving the ecological complexity often lost with single-strain. Given that IFN-γ+ CD8 T cells play a crucial role in antitumor immunity and affect ICI therapies (104), Tanoue et al. identified a consortium of 11 bacterial strains from healthy human donor feces capable of inducing IFN-γ+ CD8 T cells in the intestine (105), crucial for anti-tumor immunity and affecting ICI therapies. Colonizing mice with this 11-strain mixture improved resistance to Listeria monocytogenes infection and enhanced ICI therapy efficacy in tumor models. These strains, mostly rare and low-abundance in the human microbiota, hold promise as effective biotherapeutics (105). Further research revealed that hypoxanthine and inosine monophosphate levels are elevated in mouse serum following inoculation with the 11-strain microbial consortium. This result is consistent with the findings of a previous study reporting that inosine, a bacterial purine metabolite, can promote TH1 activation through adenosine receptor signaling, which improves the activity of ICIs in several mouse models with different types of cancer (106). *Bifidobacterium pseudolongum* and *A. muciniphila* can produce hypoxanthine and xanthine and other related metabolites, are also elevated in the serum of mice colonized with *B. pseudolongum*. An orally administered live biotherapeutic product consisting of the above 11 distinct commensal bacterial strains (VE800) was manufactured under Good Manufacturing Practice conditions and has enrolled approximately 56 patients with melanoma, gastric/gastroesophageal junction adenocarcinoma, or microsatellite-stable CRC to evaluate safety and tolerability of VE800 in combination with nivolumab into phase I-II clinical testing (NCT04208958).

Microbial ecosystem therapeutic 4 (MET4) -ICI is an investigator-initiated clinical trial conducted at a single center. It aims to assess the safety, tolerability, and engraftment of MET4 in 40 patients with advanced solid tumors undergoing ICI therapy. MET4, an alternative to fecal transplantation, is a microbial ecosystem therapeutic administered orally. It consists of 30 live intestinal bacteria cultures from a cancer-free donor. The study found MET4 to be safe and tolerable for patients on standard ICI therapy, with no severe adverse events (AEs) or increased ICI-associated irAEs. Furthermore, MET4 modified the gut microbiota and serum metabolome in ICI-naive patients, boosting taxa linked to positive ICI responses, such as *Enterococcus*, *Bifidobacterium*, and *Phascolarctobacterium*. While the first-in-human trial was not designed to evaluate ICI efficacy conclusively, it highlighted MET4’s potential to enhance ICIs’ effectiveness in treating diverse advanced-stage solid tumors (107).

MCGRAW, a multicenter, randomized, blinded, and placebo-controlled phase 1b study, focused on patients with advanced melanoma. It investigated SER-401, a microbiome therapeutic enriched with Ruminococcaceae and other spore-forming microbes. Participants were assigned to either SER-401 or a placebo, with a 2:1 ratio, and grouped based on initial Ruminococcaceae levels in their fecal. The active arm showed microbiome engraftment, though not at the desired speed or scale. The study revealed that SER-401 combined with anti-PD1 is safe for patients with advanced melanoma but noted a lower disease control rate (DCR) in the SER-401 group compared with the placebo, possibly affected by vancomycin pre-treatment. Early termination was due to enrollment issues and suboptimal engraftment of SER-401 (108).

Bacterial consortia present a promising alternative to FMT for modulating gut microbiota, offering reduced risks and improved ecological complexity. However, several limitations persist, such as the need for consistent safety and tolerability assessments in therapies like MET4 and SER-401, both of which showed initial promise but face challenges with microbial engraftment and patient enrollment. Notably, SER-401’s efficacy in advanced melanoma was compromised by lower disease control rates, potentially influenced by prior antibiotic treatments. Future research should focus on conducting larger, multicenter clinical trials to validate the safety and efficacy of these microbial therapies. Additionally, optimizing bacterial consortia for specific cancer types will be essential, along with exploring the underlying mechanisms by which these microbial treatments enhance anti-tumor immunity. This approach may ultimately lead to more effective strategies for harnessing the gut microbiome to improve cancer therapy.

### 3.3 Probiotics

Probiotics are live microorganisms that offer health benefits to the consumer when taken in adequate amounts. These benefits include altering the gut’s environment, modifying its metabolome, and interacting with the immune system (109). Commonly available probiotics, like those containing *Bifidobacterium* and *Lactobacillus* species, are recognized as safe and have demonstrated anti-inflammatory effects in the gut (110). A randomized, parallel-control study explored the biological effects of CBM588, a probiotic containing *Clostridium butyricum*, in combination with nivolumab/ipilimumab on patients with metastatic RCC (NCT03829111). CBM588 is a bifidogenic live bacterial product containing *Clostridium butyricum*, a butyrate-producing anaerobic spore-forming bacterium. In this phase I trial, 30 patients who had not received previous treatment were divided into two groups: one group received nivolumab and ipilimumab with CBM588, while the other group received nivolumab and ipilimumab only. The results showed that CBM588 increased the presence of *Bifidobacterium* spp. and led to a significantly longer PFS than those receiving nivolumab/ipilimumab only (12.7 months versus 2.5 months, P = 0.001) (111). Hibberd et al. investigated the role of microbial composition in patients with CRC and the potential of probiotics to modify the colonic microbiota. Specifically, when compared with the control group, some differences were observed in the fecal microbiota of patients with cancer, with increased microbial diversity and enrichment of *Clostridium*, *Bacteroides fragilis*, and *Enterococcus*. However, patients receiving probiotic therapy showed increased abundance of beneficial butyrate-producing bacteria such as *Faecalibacterium* and *Clostridium cluster XIVa*, indicating the potential for oral probiotics to alter the TME and serve as a potential tool in cancer treatment (112).

However, the clinical efficacy of probiotics remains a subject of debate. A study led by Spencer found that administering probiotics to mice could impair their response to ICI-based therapy. This was attributed to a reduced presence of interferon-γ–positive cytotoxic T cells in the TME (113, 114). Consequently, high doses of probiotics might be harmful. It appears that maintaining a balanced and diverse gut flora is more crucial than simply ingesting specific “beneficial” bacteria. Furthermore, the range of probiotic strains available is limited, as many beneficial species are difficult to culture or administer. These factors should be considered when considering probiotics as an adjunct to ICI therapy. Firstly, the effect of probiotics on the microbiome’s composition does not mimic the ecological benefits of FMT in those with low microbial diversity and is linked to a reduction in gut microbiome diversity compared with no treatment or FMT (115). Secondly, no single species has been consistently identified as beneficial across studies (116). Precision medicine approaches in probiotic interventions may improve their reproducibility and efficacy by enhancing the ability to modify the microbiota and boost immune responses. The development of next-generation probiotics, including microbes such as *F. prausnitzii* and *A. muciniphila*, which were previously restricted by their complex growth requirements, is promising. Future strategies will involve algorithms that use personal data and known factors affecting probiotic effectiveness to identify the best probiotic approach for specific populations or individuals (117, 118). Several factors, such as pH, H_2_O_2_, organic acids, oxygen, and moisture stress, have been identified as influencing the viability of probiotics, particularly in dairy products such as yogurt (119).

Probiotics (such as Bifidobacterium and Lactobacillus) show potential in modulating gut health and improving immune function, but their clinical efficacy remains contentious. High doses of probiotics may weaken the effectiveness of immune checkpoint inhibitor therapy, and the availability of beneficial strains is limited and difficult to manage. Moreover, the ecological benefits of probiotics are hard to compare with FMT, and there is no consistently beneficial single bacterial species. Future research should focus on applying precision medicine to probiotic interventions, developing next-generation probiotics, and optimizing their viability to identify the best probiotic approaches for specific populations.

### 3.4 Prebiotics

Prebiotics are nutrient supplements, typically non-digestible dietary fibers such as inulin, pectin, and fructooligosaccharides from fruits and vegetables (120), supporting the growth of beneficial bacteria in the gut. They enhance the gut microbiome by selectively promoting the growth and activity of commensal microbes, such as *Lactobacillus* and *Bifidobacterium*, primarily through the production of short-chain fatty acids (SCFAs). SCFAs have been shown to protect against various diseases by improving gut epithelial integrity, modulating metabolism, and stimulating immunity (121). Studies using mouse tumor models have demonstrated that oral administration of inulin gel can effectively modulate the gut microbiome, induce systemic memory T-cell responses, and enhance the anticancer activity of ICIs. Inulin-gel treatments increased the levels of key commensal microorganisms and SCFAs, leading to improved responses from interferon-γ+CD8+ T cells and the development of stem-like T-cell factor-1+PD-1+CD8+ T cells within the TME (122). Additionally, diets enriched in pectin have been shown to increase IFN-I production, alter intratumoral mononuclear phagocytes, and control tumor growth and ICI efficacy (123). Pectin can be hydrolyzed by several bacterial taxa (e.g., *Faecalibacterium, Enterococcus hirae*, and *Bacteroides fragilis*) to enhance the response to immune checkpoint blockade in patients (124). However, prebiotics such as inulin, pectin, and oligofructose can lead to primary liver cancer in 40% of genetically modified mice with imbalanced gut microbiota, and even in wild-type mice fed a high-fat diet supplemented with these prebiotics (125). This indicates that the anticancer adjuvant effect of prebiotics relies on a healthy baseline gut microbiota and metabolic context.

Recent studies have shown promising results in the field of emerging prebiotics through mouse models. Castalagin, extracted from camu-camu berries, directly interacts with the outer membrane of Ruminococcaceae spp., especially *Ruminococcus.* This interaction encourages their growth, leading to an increase in CD8+T cell activity and improved efficacy of anti-PD1 treatments (126). Similarly, ginseng polysaccharides have been found to enhance intestinal metabolism and positively affect the gut microbiota, particularly boosting the growth of *Lactobacillus* spp. and *Bacteroides* spp. These bacteria play a key role in metabolizing ginsenosides (127). Furthermore, combining ginseng polysaccharides with anti-PD-1 antibodies has shown to enhance effects by promoting IFN-γ production through CD8+ T cells activation, reducing Foxp3+ regulatory T cells in the TME, and lowering the kynurenine/tryptophan ratio in mouse models (53). Additionally, integrating anti-PD-1-based immunotherapy with the Chinese medicinal recipe Gegen Qinlian decoction effectively eliminated CRC in mice. This was achieved by altering the gut microbiota, characterized by a significant presence of *Bacteroides acidifaciens*, and by increasing levels of CD8 T cells and IFN-γ (128). Therefore, the binding of prebiotics to anti-PD-1 antibody promotes IFN-γ production through CD8+ T-cell activation, ultimately enhancing the antitumor effect.

Prebiotics like inulin and pectin can support beneficial gut bacteria but pose risks, as they may lead to primary liver cancer in certain mouse models with imbalanced gut microbiota. Their anticancer efficacy relies on a healthy gut environment. Future research should explore emerging prebiotics, such as castalagin from camu-camu berries and ginseng polysaccharides, which enhance immune responses and improve anticancer treatments. Studies should aim to optimize these prebiotics for specific beneficial bacteria while focusing on maintaining a healthy gut microbiome to maximize therapeutic benefits in combination with immunotherapies.

### 3.5 Synbiotics and postbiotics

Synbiotics, which are combinations of probiotics and prebiotics working together, offer enhanced health benefits to the host (119). The probiotics in synbiotic formulations typically include strains such as *Lactobacillus*, *Bifidobacteria* spp., *S. boulardii*, and *B. coagulans*, while the prebiotics mainly consist of oligosaccharides such as fructooligosaccharide, galactooligosaccharide, xylooligosaccharide, inulin, and naturally sourced prebiotics from chicory and yacon roots, among others. Synbiotics may offer a more effective alternative to prebiotics by enhancing microbial diversity and addressing some limitations of single-strain probiotics. The potential health benefits of synbiotics for humans include improved immunomodulatory functions and enhanced liver function in patients with cirrhosis (129). Currently, several clinical trials are exploring the effectiveness of synbiotic interventions in enhancing responses to checkpoint blockade therapies across various cancer types (NCT05032014, NCT04699721, and NCT03829111) (130).

Microbes are also recognized for their role in modulating antitumor immunity through metabolite production. Postbiotics, as defined by Tsilingiri et al., comprise substances released by or produced through the metabolic activity of microorganisms, which have a beneficial effect on the host, either directly or indirectly (131). They offer health benefits through mechanisms similar to those of probiotics but without the risks associated with live microorganisms, thereby overcoming some challenges related to microbial heterogeneity. Postbiotics could be valuable adjuvants in immunotherapy treatments involving ICIs. For instance, EO2401 is an innovative cancer peptide vaccine based on similarities between TAAs and peptides derived from the microbiome. It is being tested alone and in combination with nivolumab and nivolumab/bevacizumab to gather preliminary safety and efficacy data in patients with progressive glioblastoma. An example of leveraging microbes to boost ICIs’ efficacy without directly targeting the microbiota is the ROSALIE trial (NCT04116658). The ROSALIE trial aims to evaluate a therapeutic vaccine consisting of gut microbiota-derived peptides designed to activate commensal-specific memory T cells that can cross-react with highly homologous tumor antigens, in addition to nivolumab treatment for patients with glioblastoma (132). Early results show significant immune responses to at least one of the three microbiota-derived peptides in nearly all participants, indicating the potential effectiveness of this approach in patients with tumors that have low neoantigen levels (12, 133).

Synbiotics, combining probiotics and prebiotics, offer enhanced health benefits but face challenges like microbial heterogeneity. Postbiotics, microbial metabolites, provide similar benefits without the risk of living microorganism. Clinical trials (NCT05032014, NCT04699721, NCT03829111) explore synbiotics’ efficacy in cancer immunotherapy. Innovative vaccines like EO2401 and ROSALIE trial (NCT04116658) aim to boost immune responses using microbiota-derived peptides. Future research should focus on optimizing synbiotics and postbiotics for specific health benefits and enhancing immunotherapy efficacy.

### 3.6 Dietary interventions

Earlier reports have suggested that altering certain nutrients in the diet can improve the effectiveness of cancer therapies, with many clinics recommending that patients follow standard healthy-eating guidelines (134). These studies primarily focus on how nutrients supplied by the host can support tumor growth and survival (135). It is increasingly evident that diet can influence the composition and function of the gut microbiota, which in turn can directly affect immune function (136, 137). As a result, dietary interventions aimed at optimizing the gut microbiota to enhance the antitumor immune response to immunotherapy have received significant attention. Clinical studies have shown that specific dietary regimens, such as a high-fiber diet, Mediterranean diet, and omega-3-rich diet, are associated with a better response to ICI (138, 139). Conversely, a Western-style diet, high in saturated fats and low in fiber, is generally considered to increase cancer risk due to dysbacteriosis and a decrease in SCFA levels (140).

#### 3.6.1 High-fiber diet

Consuming a high-fiber diet has been shown to offer significant protection against colorectal and breast cancer (141). An observational study found that melanoma patients on high-fiber diets were about five times more likely to respond to immunotherapy than those on low-fiber diets. This response was linked to greater microbial diversity and an abundance of fiber-fermenting microbes, such as *F. prausnitzii* (114). Further research by the same team revealed that higher dietary fiber intake was associated with significantly improved PFS in 128 patients with melanoma on ICIs, especially in those with sufficient dietary fiber intake and no probiotic use. Patients with adequate fiber intake also had higher microbial diversity and higher abundances of the family Ruminococcaceae and genus *Faecalibacterium*. In mice, a fiber-rich diet led to delayed tumor growth compared to a fiber-poor diet when treated with anti-PD-1, suggesting that high-fiber diets can enhance the effectiveness of ICIs (113). Indigestible dietary fiber, fermented by anaerobic bacteria in the colon such as *Clostridium*, *Bifidobacterium*, and *Lactobacillus* spp., produces SCFAs such as acetate, propionate, and butyrate. These metabolites are vital for promoting epithelial cell renewal and maintaining gut barrier integrity, which helps limit systemic inflammation (142, 143). SCFAs have also been shown to influence the function of various immune cell populations, including Tregs, effector T cells, and γδT cells (144–147). Further studies have demonstrated that the SCFA butyrate enhances ICI responses by inhibiting histone deacetylase activity in CD8+ T cells and inducing expression of the inhibitor of DNA binding 2, which boosts T cell activation and reduces exhaustion (148). However, the link between SCFAs and clinical responses to ICIs in patients with cancer remains contentious. A study involving 52 patients with solid tumors undergoing PD-1 blockade treatment found that high concentrations of SCFAs were associated with longer PFS (149). Yet, high blood levels of butyrate were linked to resistance to CTLA-4 blockade and a higher proportion of Treg cells (40). These findings highlight the importance of considering various factors, such as the baseline diet, microbiota, and treatment history, to determine the effectiveness of dietary interventions (39). Notably, the most significant changes in gut microbiota were observed in patients with the lowest baseline fiber intake, suggesting that high-fiber diet interventions might benefit those who previously did not meet recommended fiber intake levels (12). However, because of the small number of patients evaluated and the variability in factors affecting SCFA production across studies, the effect of SCFA supplementation on ICI efficacy remains unclear. Ongoing RCTs are exploring the efficacy of a high-fiber diet in improving responses and outcomes to checkpoint inhibitors across multiple tumor types (NCT04645680, NCT04866810, and NCT04866810) (12).

#### 3.6.2 Ketogenic Diet (KD)

The KD, characterized by high fat, moderate protein, and low carbohydrate intake, has been shown to inhibit cancer progression (135). In mouse models, KDs alter the gut microbiota composition (150), leading to systemic ketosis. This metabolic state increases the abundance of bile-tolerant Bacteroidetes (e.g., *Alistipes* spp., *Bilophila* spp., and *Bacteroides* spp.) and decreases butyrate-producing Firmicutes (e.g., *Roseburia* spp., *Eubacterium rectale*, and *Ruminococcus bromii*) in humans (151–153). Specifically, in CRC mouse models, a ketogenic diet increased the presence of commensal *Eisenbergiella massiliensis*, correlated with higher serum levels of the ketone body 3-hydroxybutyrate and induce T-cell based antineoplastic effect in a 3HB-dependent manner, thereby promoting ICI efficacy. This increase (154, 155) induced a T-cell-mediated anticancer effect, enhancing the efficacy of ICI and improving survival rates. Interestingly, the antitumor effect of KDs seems to be independent of the microbiota, as antibiotics did not affect tumor suppression, suggesting a direct stimulation of the immune system (154, 155). A clinical trial is exploring the efficacy of KD, with variations in diet scheduling or β-hydroxybutyrate supplementation, in combination with nivolumab and ipilimumab in patients with metastatic RCC (NCT05119010) (12).

Additionally, calorie restriction (CR) in mice has been found to enrich *Bifidobacterium* in the gut microbiome. Administration of *Bifidobacterium bifidum* alone can mimic the anti-tumor effects of CR in microbiota-depleted mice through acetate production, dependent on the presence of interferon-γ+CD8+ T cells in the TME (156). However, caution is advised in applying CR to modulate the gut microbiota in advanced cancer patients with cachexia. MicrSoy-20 (MS-20), a fermented soybean extract, has been approved for alleviating chemotherapy-associated fatigue and appetite loss by modulating the gut ecosystem and immunity (157). It also acts as an anti-PD-1 booster by activating TILs, especially enhancing their migration and presence in tumors. A RCT assessed the safety and potential clinical outcomes of combining pembrolizumab and MS-20 (NCT04909034) in 30 patients with NSCLC. Moreover, long-term ethanol exposure decreases the abundance of butyrate-producing Clostridiales (e.g., *Faecalibacterium prausnitzii*, *Coprococcus eutactus*), while abstaining from alcohol restores gut barrier integrity (158, 159). This highlights the importance of lifestyle and behavioral interventions, such as dietary changes, reducing alcohol consumption, quitting smoking, engaging in physical activity, and controlling environmental pollutants, in potentially enhancing cancer therapy efficacy and improving patient quality of life.

Dietary interventions have shown promise in enhancing cancer therapy effectiveness, particularly through their impact on gut microbiota and immune function. While studies indicate that diets rich in fiber or Mediterranean-style foods improve immune responses to immunotherapy, Western diets high in saturated fats may increase cancer risk due to dysbiosis and reduced SCFAs. However, the link between SCFA levels and clinical responses to ICIs remains inconsistent, indicating the complexity of dietary effects. The KD has demonstrated antitumor effects and changes in gut microbiota but may also work independently of microbiota. Ongoing randomized controlled trials are examining the impacts of these dietary regimens on ICI treatment outcomes. Future research should explore individualized dietary approaches considering patients’ baseline diets and microbiomes to optimize dietary interventions in cancer therapy.

## 4 Outlook

The studies discussed highlight the significance of understanding the influence and regulation of gut microbiota on the success of immunotherapy treatments, offering promising directions for enhancing antitumor immunotherapy. Evidence suggests that individuals with specific gut microbiota profiles may benefit more from immunotherapy. Despite notable differences in gut bacterial composition across various regions or ethnicities due to diet and lifestyle, certain bacterial taxa, including *Akkermansia muciniphila* (16), *Bifidobacterium longum* (15), and *Faecalibacterium* spp (17), have been consistently associated with improved responses to ICI therapy. This implies that analyzing patients’ fecal samples to examine the microbiota composition could help predict the effectiveness of ICIs treatments.

Microbiota research is immensely diverse, yet the technological limitations in this field mean that evidence supporting clinical applications remains inadequate. For example, current studies reveal inconsistencies, such as no universal predictive value in individual microbial taxa or α-diversity for treatment response. These discrepancies may be attributed to various factors including clinical diversity (such as disease stage, type of immunotherapy, and treatment history), response definitions, cancer types, geographic differences, and small study cohorts. Technical aspects such as sample collection and storage can also impact the intestinal flora. Future research should thoroughly document potential confounders, increase sample sizes, and incorporate stratification and adjustment for confounders in both design and analysis phases (160). Interestingly, the observed varied associations between specific bacterial taxa and immunotherapy response may highlight the functional redundancy among taxa, suggesting that models based on microbial gene functions might offer improved predictive accuracy (161). The creation of synthetic microbial communities represents a promising avenue for precise adjunct therapies (162). However, technological challenges, such as standardizing sampling and analysis methods and establishing validation cohorts, complicate the translation of microbial biomarkers to clinical settings. Notably, the low microbial biomass in many tumor-associated ecological niches and the issue of DNA contamination present significant hurdles in both polymerase chain reaction based 16S rRNA gene surveys and shotgun metagenomics (163, 164). Therefore, when profiling the microbiome, multiple measures must be implemented to avoid any possible contamination, such as adding negative and positive sequencing controls, randomizing samples and treatments, critically assessing and reporting contributions of contamination during analysis (165). While some studies suggest that changes in the abundance of specific strains can predict immunotherapy outcomes, others find that a balanced or rich microbiome rather than dominant growth of specific “beneficial bacteria” is a better predictor of immunotherapy efficacy. For instance, a study on patients with NSCLC undergoing immunotherapy showed that while the presence of *A. muciniphila* in the gut microbiota was beneficial, a relative abundance of *A. muciniphila* greater than 5% had a negative effect on treatment response (52). Additionally, preliminary findings suggest that the effectiveness of ICIs in previously resistant patients depends on successful microbial engraftment (96, 97), highlighting the importance of optimizing engraftment predictions (12).

Research in both laboratory and clinical settings has uncovered how the gut microbiota influences antitumor immunity. This involves interactions between microbial components or byproducts, such as pathogen-associated molecular patterns, with antigen-presenting cells (APCs) and innate immune effectors (through pattern-recognition receptors such as Toll-like receptors). This interaction is pivotal for initiating an adaptive immune response. Furthermore, the stimulation of cytokine release from APCs or lymphocytes has been identified as critical components of this modulatory mechanism (166). Another mechanism is through metabolite production. Additionally, the role of cytokines released from immune cells in this process is crucial. Metabolites produced by certain gut bacteria, such as inosine from *A. muciniphilia* and *Bifidobacterium pseudolongum*, enter the bloodstream and support immune responses by Th1 activation, enhancing the effectiveness of ICIs (106). Other metabolites including SCFAs and anacardic acid also (49) play a role in fighting tumors. Understanding how the gut microbiota and its metabolites directly and indirectly affect cancer can lead to better responses to ICIs (167). However, the exact mechanisms by which the gut microbiota influences the immune system still need clarification.

## 5 Conclusion

This study reviews the role of gut microbiota in tumor immunotherapy, with a particular emphasis on its potential as a predictive biomarker for the efficacy of immune checkpoint inhibitors (ICIs). The findings indicate that specific gut microbiome signatures are positively associated with treatment outcomes across various cancers, supporting the prospect of using gut microbiota as predictors of ICI response. However, the study also highlights several limitations, including small sample sizes, unclear mechanisms, and the absence of standardized biomarkers. Future research is essential to expand sample sizes, further investigate the mechanisms underlying the interactions between microbes and immune responses, and develop personalized treatment strategies aimed at optimizing microbiome regulation to enhance the clinical effectiveness of immunotherapy. Furthermore, the role of intestinal fungi in tumor therapy, as an emerging factor in immunotherapy, warrants additional investigation. Through these efforts, we can gain a deeper understanding of the complex relationship between gut microbiota and tumor immunotherapy, ultimately leading to more effective treatment options for patients.
